# Evaluation of Somatropin Release from Chitosan and Methylcellulose Hydrogels: Influence of Hydrogel Composition and Phosvitin on the Release Profile

**DOI:** 10.3390/polym18010086

**Published:** 2025-12-28

**Authors:** Wioletta Siemiradzka, Wojciech Mizgała

**Affiliations:** Department of Pharmaceutical Technology, Faculty of Pharmaceutical Sciences in Sosnowiec, Medical University of Silesia, Jedności Street 8b, 41-200 Sosnowiec, Poland; s83427@365.sum.edu.pl

**Keywords:** chitosan, methylcellulose, somatotropin, phosvitin, biodegradable hydrogels, rheological properties, texture analysis, release testing, prolonged release, skin application

## Abstract

This article attempts to develop hydrogel systems containing two polymers, chitosan and methylcellulose, with somatotropin as the active substance. The aim of the study was to obtain a hydrogel preparation for the skin that releases a sufficient amount of the hormone to achieve a satisfactory therapeutic effect. This innovative method of delivering the hormone to the body would allow for non-invasive administration of the drug, which would certainly ensure greater comfort for patients. The preparations were subjected to an assessment of physicochemical parameters such as pH measurement, texture analysis, rheological properties, and sensory evaluation. Somatotropin release studies demonstrated that the highest hormone release occurred from a matrix containing equal amounts of chitosan and methylcellulose, reaching 2%. The use of phosvitin as a carrier protein resulted in a prolonged release of somatotropin. Among the phosvitin concentrations tested, the formulation containing 0.005% phosvitin demonstrated the highest somatotropin availability. All preparations had good rheological and textural properties, allowing them to be easily spread over the skin surface.

## 1. Introduction

Significant advances in molecular biology, peptide chemistry, and peptide delivery technologies have substantially contributed to progress in the discovery, production, and therapeutic application of peptide-based drugs. In recent years, peptides have emerged as a distinct class of therapeutic agents due to their unique biochemical properties, high therapeutic potential, relatively low immunogenicity, and reduced production costs compared with large biological drugs. The integration of peptide discovery techniques with rational molecular design has enabled the mitigation of several intrinsic limitations of peptides—such as poor membrane permeability and limited in vivo stability—thereby improving both their stability and therapeutic efficacy [1,2].

The introduction of recombinant growth hormone (GH) into clinical practice significantly expanded therapeutic possibilities owing to its unrestricted availability. This development led to the establishment of new pediatric and adult indications, with daily administration becoming the standard treatment regimen. Growth hormone, also known as somatropin, is widely used in children with growth hormone deficiency (GHD) to stimulate growth. Current clinical guidelines recommend subcutaneous administration of somatotropin (STH) once daily. Clinical evidence indicates that children with GHD who demonstrate high adherence to daily STH injections exhibit significantly better growth outcomes than those with poor compliance, which is often associated with missed doses [3].

In 2021, long-acting growth hormone (LAGH) formulations entered the global pharmaceutical market, offering improved convenience and slightly enhanced efficacy compared with daily injections. Medicine is currently entering a new phase of LAGH therapy, which is now available for both pediatric and adult patients. This therapeutic approach is expected to become predominant in the coming years, primarily due to improved patient adherence and the potential for enhanced clinical outcomes. The growing body of safety and efficacy data further supports the clinical relevance of LAGH formulations [4]. Phase II and III clinical trials have demonstrated that once-weekly STH administration is non-inferior to daily injections with respect to efficacy, safety, and tolerability, while significantly reducing treatment burden [5].

Beyond the treatment of growth hormone deficiency, growth hormones have also demonstrated therapeutic potential in wound healing applications. Pita-Vilar et al. incorporated STH into wound dressings based on carboxymethylcellulose (CMC) and silk fibroin (SF) for the treatment of chronic wounds, particularly diabetic ulcers. The inclusion of SF and GH significantly enhanced cell proliferation, migration, and angiogenesis in vitro. Moreover, GH contributed to a more balanced inflammatory response, increased antioxidant activity, enhanced vascularization, and improved tissue remodeling [6].

Nazerian et al. developed STH-containing hydrogels based on chitosan (CH) and CH/poly(vinyl alcohol) (PVA) mixtures (70/30 and 80/20) and evaluated STH release from these systems. After 1 h and 45 min, 0.014 µg/mL of somatotropin was released. The release profile was characterized by an initial burst effect attributed to drug molecules located near the hydrogel surface, followed by sustained release from the polymer matrix. The cross-linked hydrogel network retained the drug at physiological temperature and exhibited favorable physicochemical properties. Importantly, the formulations demonstrated no cytotoxicity and supported fibroblast proliferation [7].

Transdermal drug delivery systems (TDDS) represent an advanced approach for administering therapeutic agents through the skin into systemic circulation. The increasing prevalence of chronic diseases underscores the need for improved delivery systems capable of controlled and sustained drug release. As a non-invasive administration route, TDDS offers several advantages over oral and injectable therapies, including enhanced bioavailability, reduced systemic side effects, and improved patient compliance—particularly among pediatric and geriatric populations. Transdermal administration bypasses gastrointestinal degradation and first-pass hepatic metabolism, thereby improving bioavailability and maintaining more stable plasma drug concentrations [8,9,10,11].

Hydrogels are among the most widely used matrices in transdermal drug delivery systems, with hydrogel-based patches attracting considerable attention in recent years due to their unique physicochemical and mechanical properties [12]. Although needle-free devices such as jet injectors offer advantages related to needle safety, the risk of cross-contamination due to interstitial fluid splashing has limited their widespread use. Consequently, reusable nozzles have been largely abandoned, and such devices are currently applied only for repeated dosing in the same patient, as exemplified by the Tjet^®^ device (now known as ZOMA-Jet® 10, Ferring Pharmaceuticals, Saint-Prex, Switzerland) for somatotropin delivery [13].

Hydrogels are three-dimensional polymeric networks capable of retaining substantial amounts of water, making them versatile platforms for topical and transdermal drug delivery. These systems may be composed of natural, synthetic, or semi-synthetic polymers. Among them, polysaccharide-based hydrogels have received particular attention due to their biodegradability, hydrophilicity, and relatively low production costs. Owing to their inherent biocompatibility and biodegradability, hydrogels serve as effective carriers for a wide range of therapeutic agents and are suitable for multiple routes of administration, including oral, ocular, subcutaneous, vaginal, and transdermal delivery [12].

A key advantage of hydrogels designed for dermatological applications is their ability to maintain a pH close to that of healthy skin, which enhances drug permeation while minimizing the risk of irritation. Their high water-binding capacity supports skin hydration and elasticity, thereby improving patient comfort during prolonged use. Additionally, favorable mechanical and rheological properties—such as elasticity, permeability, and viscoelasticity—contribute to their functionality as advanced semi-solid drug delivery systems [14,15].

Recent studies on hydrogel formulations containing polypeptide-based active substances, including somatotropin, corticotropin, and albumin, have confirmed their ability to penetrate natural biological membranes such as the skin, intestine, and pericardium. Albumin, in particular, has been shown to enhance the stability of peptide drugs and has been increasingly used in combination with biologics to improve bioavailability and prolong therapeutic action [16,17,18]. The incorporation of specific peptides has been demonstrated to stabilize proteins and extend both their release and permeation times. These profiles depend on the preparation method and the type of hydrogel matrix employed, with previously investigated systems including sodium alginate (4%), methylcellulose (MC, 4–6%), chitosan (4%), and starch-based hydrogels containing glycerol [19,20,21,22].

Hydrogel matrices also limit protein mobility, thereby preserving the structural integrity and biological activity of encapsulated therapeutic proteins. Upon release, proteins retain their native conformation, as hydrogels provide superior protection against denaturation and aggregation compared with other matrix systems [23]. The diversity of amino acid sequences further enables precise tuning of supramolecular interactions, facilitating the design of hydrogels with customizable physicochemical and mechanical properties [24,25].

Chitosan and methylcellulose were selected for the present study due to their biocompatibility, biodegradability, and ability to form stable polymeric matrices. Although both polymers have been extensively studied, prior research has primarily focused on basic physicochemical properties or single-component systems. Hydrogel formulations containing CH and MC in various ratios were therefore developed and subsequently loaded with STH.

The MC concentration (3%) was selected based on previous studies demonstrating high STH release from 4% and 6% MC matrices, with higher concentrations leading to reduced diffusion [22]. Chitosan was used at concentrations of 1% and 2%, as higher levels may impair spreadability and hinder STH diffusion. Enoch et al. reported that hydrogels containing 0.5–2.5% chitosan show considerable potential for biomedical applications, including tissue engineering and wound dressing development, with thixotropic behavior supporting efficient structural recovery after application [26].

Chitosan is a natural polysaccharide obtained by deacetylation of chitin derived from crustacean shells, insect exoskeletons, or fungal cell walls. Its advantageous properties include bioadhesion, biodegradability, high biocompatibility, antibacterial activity, and strong gel-forming capability. Consequently, chitosan is widely employed in biomedical applications, supporting cell adhesion and proliferation and exhibiting beneficial effects in wound healing, tissue engineering, and drug delivery systems [27,28,29,30,31,32,33,34].

Hydrophilic and hydrophobic characteristics of drugs significantly influence their release behavior from hydrogel matrices. Hydrogels with high swelling capacity facilitate the release of hydrophilic drugs [35]. Key features of chitosan—such as pH-responsive gel formation, biocompatibility, and penetration-enhancing properties—make it particularly suitable for controlled-release systems [36].

The incorporation of chitosan–gallol (CH–GA) into MC hydrogels has been shown to enhance network integrity. The resulting MC/CH–GA double-network hydrogels exhibited significantly improved mechanical strength, tissue adhesion, and biological activity in vitro [37].

Stimuli-responsive cellulose-based hydrogels are extensively studied for biomedical applications due to their sensitivity to pH and temperature. Methylcellulose (MC), a methoxyl-substituted cellulose derivative, forms biocompatible, film-forming hydrogels widely used in pharmaceutical formulations for controlled drug delivery and tissue engineering [37,38]. MC-based in situ hydrogels enable sustained drug release owing to their cross-linked structure, viscosity, and mechanical strength, while chitosan may further enhance drug permeation by transient modulation of epithelial tight junctions [39,40].

Despite these advantages, the literature lacks comprehensive analyses of synergistic CH–MC systems with respect to controlled STH release and mechanical property optimization. There remains a clear need to develop optimized hydrogel carriers for protein therapeutics that provide peptide stabilization, formulation stability, high bioavailability, and therapeutic efficacy with minimized adverse effects. Owing to its favorable physicochemical and biological properties, chitosan was selected to develop an STH formulation with enhanced bioavailability compared to previously reported systems. Phosvitin was incorporated for the same purpose, as a functional excipient intended to further modulate drug stability and release.

The novelty of the present study lies in the application of defined CH/MC combinations at specific concentrations and the evaluation of previously unexplored properties of CH/MC systems, including rheological behavior, release kinetics, mechanical resistance, and formulation stability.

The incorporation of phosvitin (PV) into topical formulations may confer additional therapeutic benefits. PV is a highly phosphorylated, amphiphilic protein derived from egg yolk that readily interacts with aqueous and lipid environments and forms stable complexes with metal ions. Owing to its strong chelating capacity, PV and its phosphopeptides inhibit Fe(II)-induced lipid oxidation and exhibit antibacterial activity, particularly against Gram-negative bacteria [41].

PV used in cosmetic and dermatological formulations protects cells against oxidative stress, inhibits enzymes involved in extracellular matrix degradation, and thereby mitigates skin aging. Its topical application has been associated with enhanced collagen synthesis and reduced melanogenesis, leading to improved skin condition [42].

PV’s ability to form complexes with high-density lipoproteins further highlights its functional versatility. Its isolation from egg yolk enables the development of biopolymers suitable for dermatological applications, including formulations intended for compromised or sensitive skin. Moreover, PV exhibits antioxidant, antibacterial, and immunomodulatory activities, supporting its potential use as a natural functional ingredient or immune-modulating agent [41,43,44,45].

Previous studies demonstrated that STH penetration through porcine skin occurred within approximately 4 h in the presence of 0.0005% PV, whereas PV concentrations of 0.001% and 0.002% prolonged penetration to 7 h, with 0.002% yielding the highest permeation [46]. Based on these findings, higher PV concentrations (0.005%, 0.0075%, and 0.01%) were investigated in the present study to further evaluate their impact on STH permeation time.

The aim of this study was to develop a hybrid carrier for somatotropin based on chitosan and methylcellulose, for the purpose of designing a new drug delivery system. To our knowledge, comparative studies within the proposed polymers with somatotropin not yet been conducted for skin application.

Accordingly, the necessary evaluation of physicochemical properties of the developed formulations, including pH, rheological behavior, texture parameters, and sensory characteristics. The release and permeation behavior of STH should be investigated in relation to polymer matrices previously employed in semi-solid dosage forms. In addition, the effect of phosvitin on the kinetics of STH release from the newly developed hydrogel matrix will be assessed.

## 2. Materials and Methods

STH somatotropic hormone, Genotropin 12—somatotropin, powder for solution for injection 12 mg (36 IU), lot number LT4821, was purchased from Pfizer Europe MA EEIG, Brussels, Belgium. phosvitin (PV) was purchased from Sigma-Aldrich, USA. Chitosan was from Sigma Aldrich (average molecular weight, deacetylation: 75–85%; viscosity approximately 200–800 cP). Methylcellulose (viscosity of 2% solution at 25 °C: 3755 mPa s) was purchased from Sigma Aldrich, Inc., St. Louis, MO, USA. Acetic acid was from Avantor Performance Materials Poland SA, Gliwice, Poland. Glycerol 85% was from Microfarm, Zabierzów, Poland. Water for the injection material was purchased from Fresenius KABI, Błonie, Poland; potassium dihydrogen phosphate came from Chempur, Piekary Śląskie, Poland; sodium chloride from Chempur, Piekary Śląskie, Poland; and disodium hydrogen phosphate anhydrous from Chempur, Piekary Śląskie, Poland. The reagents used were analytical grade. The Spectra/Por^®^2 cellulose dialysis membrane, Biotech CE, with a pore size of 100 kDa, was purchased from Spectrum Laboratories Inc. (Repligen Corporation, Waltham, MA 02453, USA).

### 2.1. Hydrogel Preparation

#### 2.1.1. Preparation of Based Hydrogels

The requisite amount of chitosan was meticulously dissolved in a 0.1 M acetic acid solution at a temperature of 50 °C, followed by thorough stirring until complete dissolution was achieved. Concurrently, a water–glycerol mixture was subjected to an elevated temperature of 80 °C. Subsequent to this, a solution of methylcellulose was introduced, and the mixture was subjected to vigorous stirring. The CH solution was then slowly introduced to the MC solution and mechanically mixed at 250 rpm using a magnetic stirrer (Fisherbrand Isotemp stirring hotplate; Thermo Fisher Scientific, Mississauga, ON, Canada) until the hydrogel thickened. The resulting hydrogels were designated as CH1MC3, CH2MC2, and CH2MC3.

#### 2.1.2. Preparations with STH and STH with PV

Somatotropin was incorporated into the three hydrogel substrates by introducing its aqueous solution (prepared in approximately 1 mL of water for injection). The final somatotropin concentration in all formulations was 0.1%. Subsequently, aqueous phosvitin solutions were introduced to the STH-loaded hydrogels in amounts corresponding to final PV concentrations of 0.005%, 0.0075%, and 0.01%.

Somatotropin and phosvitin solutions were introduced into the hydrogels cooled to room temperature, with the volume adjusted by reducing the amount of water used in the methylcellulose phase. This approach ensured that the final weight of the formulations and the concentrations of STH and PV remained constant. After introduction, the preparations were gently mixed to avoid the formation of air bubbles.

Formulations containing somatotropin were designated as STH/CH1MC3, STH/CH2MC2, and STH/CH2MC3. Formulations containing both somatotropin and phosvitin were labeled as STH/CH1MC3 + PV 0.005, STH/CH1MC3 + PV 0.0075, and STH/CH1MC3 + PV 0.01. All hydrogel preparations were stored at 4 °C. The compositions of the prepared hydrogel formulations are presented in Table 1.

### 2.2. Somatotropin Release Study

To study the release of STH from the prepared hydrogel matrix, we used a white borosilicate glass Franz cell flow cell with a Type 03, 9 mm stirrer, a Type 02, 5 mL jacket, and a flat junction, supplied by LPP Equipment Warsaw (Warszawa, Poland). A three-station Franz cell stirrer with an anodized aluminum holder from LPP Equipment Warsaw and a recirculation thermostat for the LPP Equipment Warsaw Franz diffusion cell system were used. The studies were conducted using a Spectra/Por^®^2 cellulose dialysis membrane from Biotech CE with a 100 kDa pore size. The membranes were cut to completely cover the surface of the diffusion chamber. According to the manufacturer’s recommendations, the membranes were soaked in water for 30 min before application of the preparation. Subsequently, approximately 0.3 g of hydrogels containing STH and hydrogels containing both STH and PV were applied to the membrane. Thereafter, the chamber was hermetically sealed to prevent evaporation of the acceptor fluid. The Franz cells were then filled with 5 mL of acceptor fluid, which was a PBS (Phosphate-Buffered Saline) with a pH of 7.4 and the following composition: sodium chloride, potassium dihydrogen phosphate, anhydrous disodium phosphate, and water. A thermostat was then turned on, set to 32 °C, to simulate skin surface temperature conditions. A magnetic stirrer was placed in each chamber to ensure constant mixing of the solution. Before the experiment, air bubbles were removed from the interior of the chambers to prevent disruption of the diffusion process. Samples of 2 mL were taken from the Franz cells at specified time intervals: after 0.5 h, 1 h, 1.5 h, 2 h, 2.5 h, 3 h, 4 h, 5 h, 6 h, 7 h, 8 h, 9 h, and 10 h. Each time the sample was taken, the missing volume was supplemented with fresh phosphate buffer, and the air bubbles were removed again.

The STH content was determined from the calibration curve described by the equation y = 0.8953x + 0.0032 (R^2^ = 0.999), where *y* represents absorbance and *x* represents concentration. The amount of STH released was measured spectrophotometrically at wavelength λ = 276 nm, using the PBS solution with pH = 7.4 as a reference. The validated method of STH measurement and release study was described in detail in the paper by Siemiradzka during the study of STH release through the skin [22]. Measurements were made using a UV-VIS Cecil CE 3021 spectrophotometer (Cecil Instruments Limited, Cambridge, UK). The average of six repeats was calculated (n = 6). The process of somatotropin release through the skin was analyzed by plotting the substance concentration (C%) in the acceptor chamber against time (t). The total cumulative amount was presented as a percentage of the applied dose to the skin surface area, Q [%] at time t.

#### 2.2.1. Comparison of Release Profiles

The release profiles of STH from the tested pharmaceutical preparations were analyzed using statistical methods recommended by the US Food and Drug Administration (FDA) and the European Medicines Agency (EMA). A model-independent mathematical approach was also used to compare the permeation profiles of the samples and the reference product using the difference factor f1 and similarity factor f2 [47]. The values of the factors f1 and f2 were calculated by Equations (1) and (2):

Difference factor:

*f*1 = [∑|*Rt* − *Tt*|∑*Rt*] × 100 (1)

Similarity factor:

*f*2 = 50 × *log*{[1 + 1*n*∑(*Rt* − *Tt*)2] − 0.5 × 100} (2)

where R_t_; T_t_: percentage dissolved of the reference and the test profiles, respectively, at time point t; n: number of sampling points.

#### 2.2.2. Release Kinetics Analysis 

The release mechanism of STH was analyzed using three different kinetic models, Equations (3)–(6):

First-order model:

(3) $$F=100\times [1-Exp\left(k_{1}xt\right)]$$

Higuchi model:

(4) $$F=k_{H}\times t_{0.5}$$

Korsmeyer–Peppas model:

(5) $$F=k_{kp}\times t_{n}$$

Peppas–Sahlin model:

F = k_PS1_ × t^m^ + k_PS2_ × t^(2 × m)^ (6)

where F, fraction [%] of drug released in time t; k_0_, zero-order release constant; k_1_, first-order release constant; k_H_, Higuchi release constant; k_kp_, release constant incorporating structural and geometric characteristics of the drug-dosage form; n, diffusional exponent indicating the drug-release mechanism; f, amount of the drug released; t, time; k_PS1_, Peppas–Sahlin release constant (constant for Fickian diffusion); k_PS2_, constant for case II relaxational mechanism; m, diffusion exponent [48].

The coefficient of determination R^2^ (the higher the R^2^ value, the better the model fit) allows us to determine which mathematical model best describes the release process and its kinetics.

### 2.3. Measurement of pH

The prepared hydrogel preparations were subjected to pH determination using the potentiometric method as outlined in the Polish Pharmacopoeia XII [49]. The measurement was performed by directly immersing a glass electrode (In Lab Expert Pro-ISM, no. 30014096, Mettler-Toledo AG, Greifensee, Switzerland) in the hydrogel preparations.

### 2.4. Evaluation of Rheological Parameters

A Lamy Rheology RM 200 Touch rotational rheometer (Lamy Rheology Instruments, Champagne au Mont d’Or, France) controlled by Rheomatic software RM 200 (software from Lamy Rheology Instruments, Champagne au Mont d’Or, France) was used to determine the rheological parameters of the prepared hydrogels. A plate-on-plate measurement system with a CP 2445 measuring spindle and a CP-1 Plus thermostat was used for the measurements. Before the measurements, the samples were removed from the refrigerator and, after 30 min, were placed on the bottom plate of the thermostatic system. The measurements were performed at a temperature close to human skin temperature: 32.0 ± 0.5 °C. Additionally the viscosity was measured at 25 ± 0.5 °C. The viscosity of the hydrogels was measured at three shear rates: D = 30 s^−1^, D = 60 s^−1^, and D = 100 s^−1^. Flow test curves were determined over a shear rate range of D = 5–200 s^−1^, with a measurement time of 34 s. The study was performed for hydrogel substrates unloaded with protein substances and for formulations containing STH and STH with the addition of PV.

Graphical and mathematical analysis of the obtained results was performed with the Rheometric-P Software RM 200. The flow curves of the analyzed hydrogels were plotted. The relations between shear stress and shear rate were analyzed using various rheological models, Equations (7)–(10):

Ostwald-de Waele:

(7) $$\tau =K\times \gamma^{n}$$

Herschel–Bulkley:

(8) $$\tau =\tau_{0}+K\times \gamma^{n}$$

Bingham:

(9) $$\tau =\tau_{0}+\eta \times \gamma$$

Casson:

(10) $$\tau^{0.5}=\tau_{0}^{0.5}+\eta^{0.5}\times \gamma^{0.5}$$

where τ, shear stress (Pa); τ_0_, yield stress or yield point; γ·, shear rate (s^−1^); K, consistency coefficient (Pa)^1/2^(s)^n^; η, viscosity (Paxs); n, flow behavior index.

### 2.5. Texture Analysis

Texture measurements belong to the group of measurements of the mechanical properties of solids and liquids, which are closely correlated with sensory tests. Texture analysis of the tested preparations was performed using a TX-700 Lamy Rheology texturometer (Lamy Rheology Instruments, Champagne au Mont d’Or, France) using the TPA Cycle and CRT functions. TPA measurement is performed by compressing the sample twice (2 cycles) and recording the force, distance, and dimensions. The test was conducted at room temperature. A cylindrical steel sensor with a diameter of 8 mm was used for the measurement. It was immersed twice in each sample at a speed of 1 mm/s, with an initial force of 0.05 N and a relaxation time of 20 s between compressions, to a depth of 10 mm. Based on the texture profiles, the hardness, cohesion, adhesion, elasticity, adhesion strength, and relaxation of the prepared hydrogels were determined. The analysis, conducted in CRT (direct compression/relaxation/tension) mode, involved measuring compression under the following conditions: compression speed, 1.0 mm/s; relaxation time (or time between cycles), 20 s; and distance, 10.0 mm. RheoTex software (firmware version: 1.37.0.0) for the TX-700, version TX-UK01/2019, was used to record and analyze the test results.

### 2.6. Sensory Evaluation

Sensory evaluation was performed using the organoleptic method, using the skin of the hand and forearm. A five-point scale was used, where 1 indicated no agreement with the assessed characteristic, and 5 indicated a strong agreement. Six characteristics were analyzed: uniformity, consistency, cushion effect, adhesion, ease of spreading, and stickiness. Uniformity was assessed by applying approximately 0.5 cm^3^ of the preparation to the skin of the forearm, then spreading it in circular motions, observing the presence or absence of lumps and air bubbles. Consistency, as an indicator of cohesion and density, was tested by immersing the finger at a 45–60° angle in a sample of approximately 20 cm^3^ and then quickly removing it. Particular attention was paid to the resistance of the substrate during this test. The cushion effect was assessed by placing 0.5 cm^3^ of the preparation between the thumb and index finger, then rubbing them together to assess the perceived amount of substance and its elasticity. Adhesion was tested by lightly touching the sample with the fingertip—the formation of a cone was an indication of good adhesion. The spreadability and viscosity parameters were assessed by applying 0.5 cm^3^ of the product to the skin of the forearm. For spreadability, the degree of resistance to spreading was determined, while stickiness was assessed based on the force with which the hand adhered to the surface of the spread gel.

### 2.7. Stability Test

The stability of the hydrogels was evaluated according to the biotechnology/biological product stability testing guidelines specified in ICH Q5C. Samples were stored for 4 weeks under controlled conditions of 25 ± 1 °C [50]. Stability assessment included analysis of sensory attributes, color, drug content, pH, viscosity, and texture parameters. The pH was measured using an InLab Expert Pro-ISM electrode (part no. 30014096, Mettler-Toledo AG, Greifensee, Switzerland). The somatotropin content was quantified spectrophotometrically at λ = 276 nm using a UV–VIS spectrophotometer (CECIL CE 3021, Cecil Instruments Limited, Cambridge, UK). Viscosity measurements were performed with a Lamy RM 200 Touch rotational rheometer (Lamy Rheology Instruments, Champagne-au-Mont-d’Or, France). Texture parameters were determined using a TX-700 texturometer (Lamy Rheology Instruments, Champagne-au-Mont-d’Or, France). All prepared formulations were found to be stable. API content was within the acceptable limit of 90% of the initial value [51]. The viscosity values measured at 32 ± 0.5 °C, as well as the texture parameters and pH after 4 weeks of storage, are presented in Tables S2–S4 in the Supplementary Materials.

### 2.8. Statistical Analysis

A statistical software program (Statistica 13.0, STATSOFT; Statistica, Tulsa, OK, USA) was utilized to conduct all statistical analyses. The experimental data were expressed as the mean (M) with the standard deviation (SD). One-way analysis of variance (ANOVA) followed by the use of Tukey’s multiple comparison tests was employed to test the difference between groups; *p* < 0.05 was considered significant.

## 3. Results

### 3.1. Release Study

**Figure 1:**
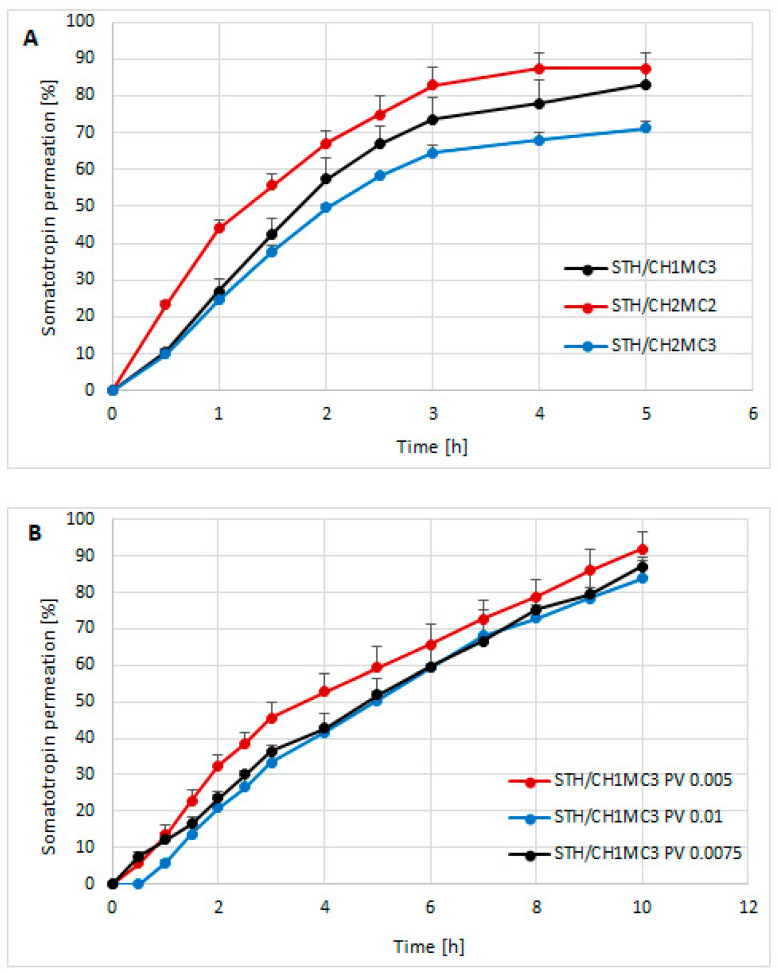
The course of the STH release process from semi-solid hydrogel preparations: STH/CH1MC3, STH/CH2MC2, and STH/CH2MC3 (**A**) and the course of the STH release process from hydrogel preparations with the addition of phosvitin (**B**). Average of 6 measurements with standard deviation.

Based on the study of STH release through a cellulose membrane of semi-solid hydrogels intended for skin application, the course of this process is graphically presented in Figure 1A,B. Figure 1A shows the amounts of STH permeated through a cellulose membrane from three different hydrogel preparations: STH/CH1MC3, STH/CH2MC2, and STH/CH2MC3. Figure 1B shows the amounts of STH permeated after introducing phosvitin at three different concentrations to the STH/CH1MC3 hydrogels: 0.005%, 0.0075%, and 0.01%.

Among the STH-containing formulations, the STH/CH2MC2 hydrogel—containing equal concentrations of chitosan and methylcellulose (2% each)—exhibited the highest STH availability. In the formulations containing 1% CH and 3% MC (STH/CH1MC3) and 2% CH and 3% MC (STH/CH2MC3), a delaying effect on STH release was observed, attributable to the higher MC content relative to the CH2MC2 formulation. The total amounts of STH released were as follows: STH/CH1MC3, 83.05 ± 8.39%; STH/CH2MC2, 87.28 ± 4.27%; and STH/CH2MC3, 71.26 ± 1.80%. The highest release (87.28% ± 4.27%) was observed for the STH/CH2MC2 preparation, with STH released within 4 h. In the case of the STH/CH1MC3 (83.05% ± 8.39%) and STH/CH2MC3 (71.26% ± 1.80%) preparations, STH release was completed after 5 h. Increasing the CH concentration from 1% to 2% while reducing the MC concentration from 3% to 2% shortened the release time (4 h) compared with the STH/CH1MC3 and STH/CH2MC3 systems (5 h). Conversely, increasing CH from 1% to 2% decreased the total amount of STH released. On the basis of these results, one model formulation was selected for further studies aimed at developing a system with prolonged release. The STH formulation based on CH1MC3 was selected due to the longer STH release time compared to the STH/CH2MC2 formulation and the higher amount of released STH compared to the STH/CH2MC3 formulation. The rheological and textural properties of the STH formulation to which PV protein was introduced also influenced the selection process. The incorporation of phosvitin into the STH formulation at all concentrations resulted in an extension of the STH release time to a minimum of 10 h. The formulation containing STH with PV at a concentration of 0.005% demonstrated the highest level of STH release, reaching 91.84% ± 4.85%.

The formulation with PV 0.0075% initially (after 0.5 h) showed approximately 1.5-fold higher release compared to the formulation containing PV 0.005%, but at subsequent time points the amount of STH released was lower, finally reaching 87.06% ± 1.74% after 10 h. The highest PV concentration of 0.01% resulted in an additional delayed release. STH was detectable in the acceptor fluid only after one hour. After 10 h, the total amount of released protein was the lowest (83.76% ± 5.82%) among all formulations tested but was similar to the amount of STH released after 5 h without PV. These results indicate that the addition of PV significantly affects the kinetics of STH release, not only prolonging the release time but also modulating the total amount of released substance, depending on the concentration used. As the PV concentration increased, a lower amount of STH release was observed in each time interval. The parameters of the STH release process, calculated using three kinetic models—Higuchi, Korsmeyer-Peppas, Peppas-Sahlin, and first-order—and the area under the curve (AUC), calculated using the trapezoidal rule, are presented in Table 2.

The determination coefficients R^2^ for STH/CH1MC3, STH/CH2MC2, and STH/CH2MC3 hydrogels are highest for the first-order kinetic model. According to the first-order model, the rate constants of the release process were as follows: the highest, 0.412 ± 0.036 for STH/CH2MC2, 0.374 ± 0.012 for STH/CH1MC3, and 0.249 ± 0.012 for STH/CH2MC3. STH demonstrated the fastest release from the CH2MC2 vehicle, which contained 2% CH and 2% MC. The release rate was determined to be 0.103 ± 0.004 mg/cm^2^ h^−1^. The augmentation of the MC content from 2% to 3% led to a reduction in the release rate to 0.087 ± 0.01 mg/cm^2^ h^−1^ (STH/CH1MC3) and to 0.075 ± 0.004 mg/cm^2^ h^−1^ (STH/CH2MC3). The presence of elevated concentrations of these polymers resulted in a protracted release of STH from the CH2MC3 base. For the unmodified formulations, the STH/CH2MC2 demonstrated the highest area under the curve (AUC) at 243.23 ± 11.55 at 4 h. For the formulations containing PV, the release kinetics were better described by the Peppas–Sahlin and Higuchi models; however, higher coefficients of determination (R^2^) were obtained for the Peppas–Sahlin model. The incorporation of phosvitin into the STH formulations extended the release time of STH to 10 h. The hydrogel containing STH/CH1MC3 + PV 0.005 had the highest AUC value (552.28 ± 43.22). Increasing the PV content to 0.0075% and 0.01% slightly decreased the AUC to 491.76 ± 5.09 and 471.62 ± 49.73, respectively. Consequently, additional increases in PV concentration did not yield substantial variations in the release rate. However, it is noteworthy that the presence of PV at a concentration of 0.01% resulted in a delay in the release process.

The release profiles of the formulation containing STH and PV differ significantly from those of the formulation with STH (Table 3). This confirms a change in the release mechanism after the introduction of PV. Among the PV-containing formulations, the release profiles of STH with 0.0075% and 0.01% PV are similar, with no statistically significant difference. The STH formulation with 0.005% PV differs significantly from the other formulations containing PV. The higher the PV concentration, the less STH is released from the hydrogel formulations. 0.005% PV appears to have the most beneficial effect on STH availability.

### 3.2. Rheological Properties

Viscosity values measured for three selected shear rates—30 s^−1^, 60 s^−1^, and 100 s^−1^ at 32.0 ± 0.5 °C are presented in Table 4. No significant differences were observed between the viscosity determined at 32.0 ± 0.5 °C and the viscosity at 25 ± 0.5 °C. The viscosity values measured at 25 ± 1 °C are presented in Table S1 in the Supplementary Materials. The hydrogel formulations prepared for the study were characterized by good viscosity within the studied shear rates. The lowest viscosity values were characterized by the base vehicles: 12,080.8 ± 120.3 mPa·s for CH2MC2, 13,674.6 ± 117.1 mPa·s for CH1MC3, and 12,378.3 ± 116.3 mPa·s for CH2MC3 at a shear rate of 30 s^−1^. The introduction of STH into the hydrogels resulted in an increase in viscosity to 14,440.5 ± 119.1 mPa·s STH/CH2MC2, 18,173.8 ± 105.9 mPa·s STH/CH2MC3, and 19,097.1 ± 321.6 mPa·s STH/CH1MC3 at the same shear rate of 30 s^−1^. In turn, PV caused a decrease in the viscosity of hydrogel preparations with STH. The viscosity of these formulations at a shear rate of 30 s was 16,663.3 ± 316.8 mPa·s for STH/CH1MC3 + PV 0.005, 15,134.7 ± 284.8 mPa·s for STH/CH1MC3 + PV 0.0075, and 17,388.3 ± 350.2 mPa·s for STH/CH1MC3 + PV 0.01. Similar relations were observed for the remaining shear rates: 60 s^−1^ and 100 s^−1^.

**Figure 2:**
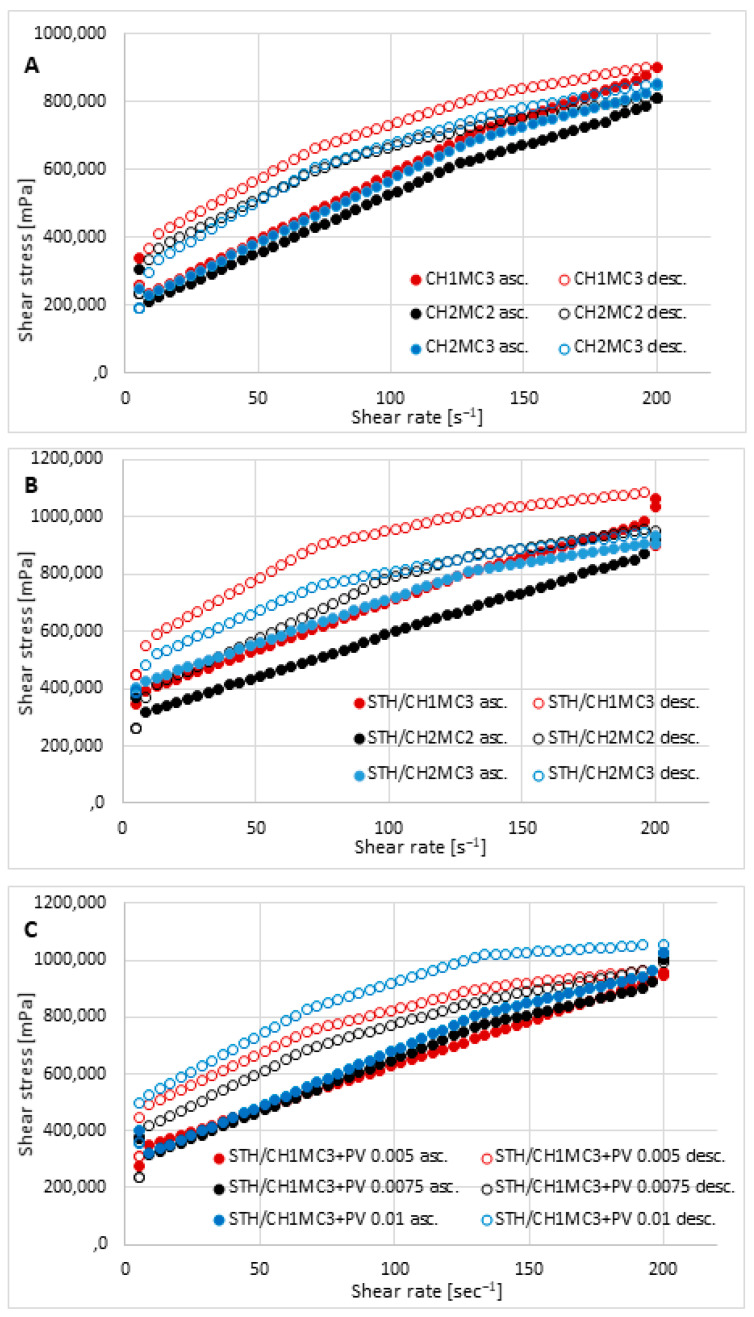
Flow behavior of the prepared hydrogels: unloaded hydrogel bases CH1MC2, CH2MC2, and CH2MCC (**A**); hydrogels with STH—STH/CH1MC3, STH/CH2MC2, and STH/CH2MC3 (**B**); and hydrogels with STH and PV—STH/CH1MC3 + PV 0.005, STH/CH1MC3 + PV 0.075, and STH/CH1MC3 + PV 0.01 (**C**); asc.—ascending curve, desc.—descending curve; mean with SD (n = 6).

It was observed that as the CH concentration increased from 1% to 2%, the viscosity decreased slightly, while higher MC concentrations (3%) caused a slight increase in viscosity. MC increased the viscosity to a greater extent than CH. The introduction of STH increased the viscosity of the base hydrogels at all shear rates, while PV increased the viscosity of the base vehicles but decreased the viscosity of the formulations containing STH. Figure 2A presents the flow curves of the hydrogel matrices unloaded with active substances. Figure 2B represents the flow curves of the hydrogels loaded with STH, and Figure 2C shows the flow curves of the hydrogels containing STH and PV. Analysis of the viscosity-shear rate relationship revealed shear-thinning behavior (pseudoplastic flow) but also a time-dependent change in viscosity in the gel phase, visible in Figure 2A–C as hysteresis rheograms. All prepared hydrogels behave like non-Newtonian fluids, belonging to thixotropic, shear-thinning systems. As shear rate increases, viscosity decreases up to a certain point, i.e., the maximum spindle rotational speed. After that, as the shear rate decreases, viscosity increases, reaching higher values at the corresponding shear rate points. The ascending part of the graph does not coincide with the descending part (which is higher), creating a characteristic hysteresis loop in the rheograms. The viscosity measured in the descending parts of the rheograms was higher than in the ascending parts for the same shear rates, and the phenomenon is called thixotropy (visible as negative hysteresis). In the case of preparations prepared on the basis of CH and MC, a time-dependent increase in viscosity was observed, i.e., “negative thixotropy”, i.e., reopexy, which has been described in the literature [52,53]. The larger the hysteresis loop area, the greater the thixotropic properties and, consequently, the greater the stability of the preparation [54,55].

The hysteresis loop areas for the prepared preparations (thixotropy), calculated using the trapezoidal method, from the highest value are as follows: STH/CH1MC3 + PV 0.01 454,197.5 Pa·s^−1^, STH/CH1MC3 443,278.5 Pa·s^−1^, STH/CH1MC3 + PV0.005 315,785.0 Pa·s ^−1^, STH/CH2MC2 306,292.0 Pa·s^−1^, CH1MC3 248,449.5 Pa·s^−1^, STH/CH1MC3 + PV 0.0075 245,653.8 Pa·s^−1^, CH2MC2 215,345.6 Pa·s^−1^, CH2MC3 116,520.6 Pa·s^−1^, and STH/CH2MC3 111,087.2 Pa·s^−1^.

Based on the parameters determined using rheological models (Table 5), it was determined based on the coefficients of determination that the rheological properties were best described by the Herschel–Bulkley equation, for which the R^2^ values were the highest. The Ostwald equations had similar, but slightly lower, coefficients of determination. Based on the Herschel–Bulkley equation, all formulations demonstrated very good flow properties and a significant degree of shear thinning, resulting in clearly pronounced pseudoplastic properties. The flow index values “*n*” were less than 1.0 and ranged from 0.105 STH/CH2MC2 to 0.448 STH/CH2MC3. In reality, the “flow index” varies from values around unity, typical of Newtonian behavior, to values between 0 and 1, characteristic of non-Newtonian systems. Any system exhibiting “melt index” values lower than 1 and “yield stress” values higher than 0 can be defined as a plastic system, which is a special case of non-Newtonian fluids [56].

The rheograms in Figure 2 indicate that the tested hydrogel formulations tested have a yield stress. The yield stress is the minimum shear stress at which the spatial structure (polymer network or physical bonds between molecules) is partially destroyed, and the material begins to deform permanently and flow. The yield stress determined using the Herschel–Bulkley model had the following values: the highest value was 0.02 Pa for the CH2MC2 and CH2MC3 formulations and 0.05 Pa for the remaining formulations. Consistency indices ranged from 81.9 (STH/CH2MC3) to 889.7 (STH/CH2MC2).

### 3.3. Texture Analysis

**Figure 3:**
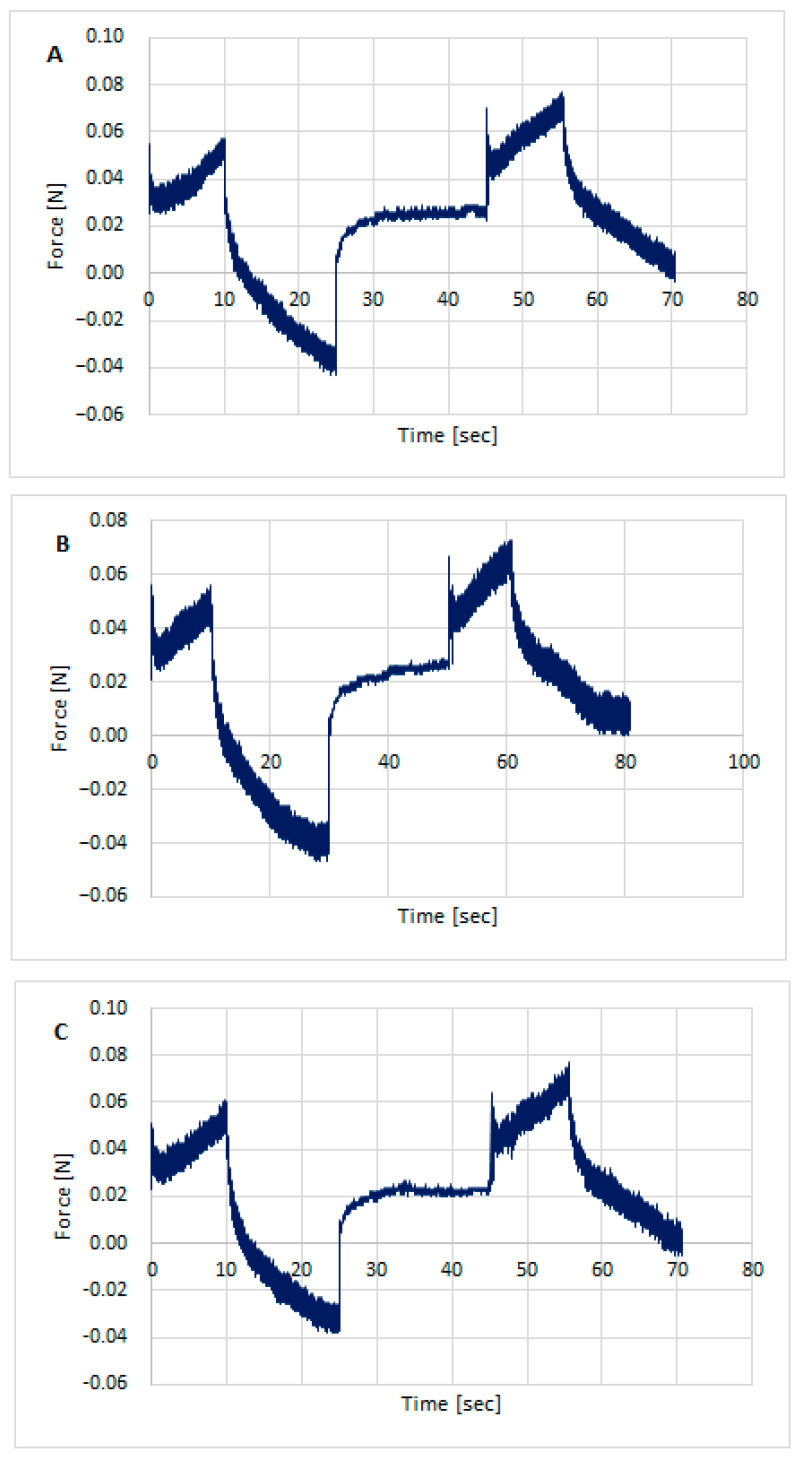
Texture profile analysis (TPA) of preparation unloaded/without the active substances: CH1MC3 (**A**), CH2MC2 (**B**), and CH2MC3 (**C**).

**Figure 4:**
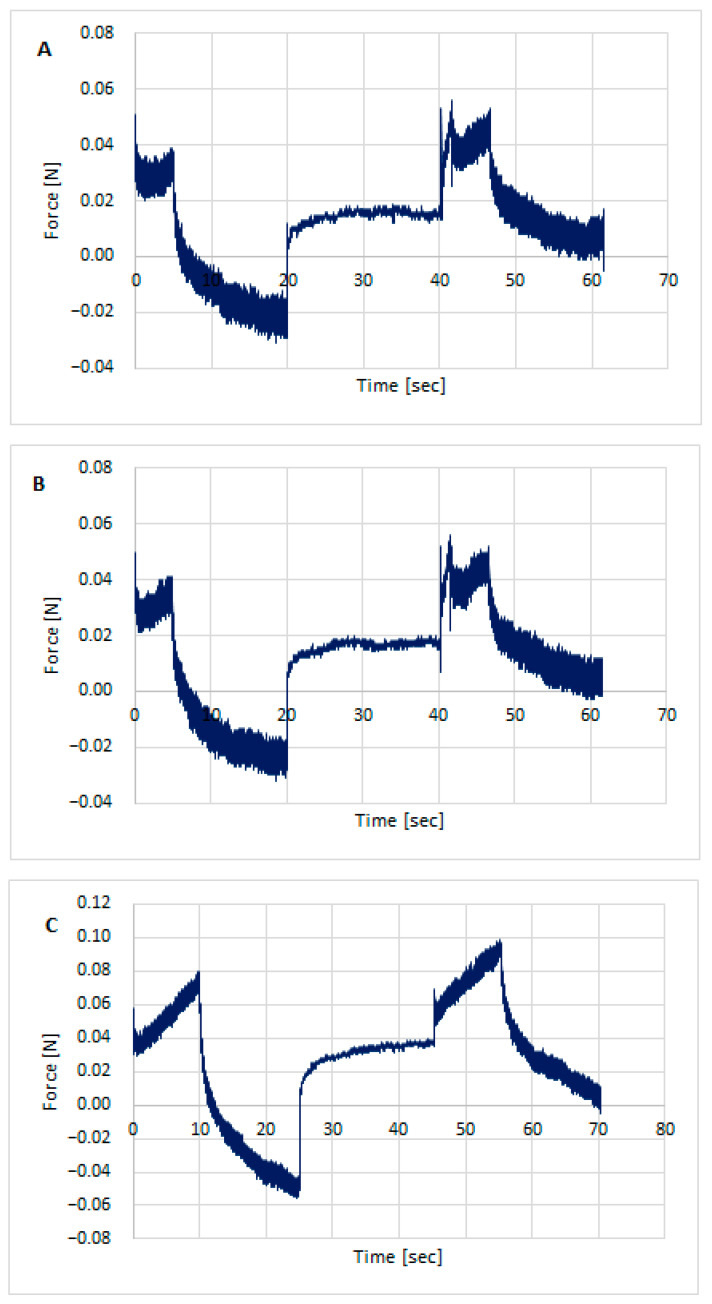
Texture profile analysis (TPA) of hydrogels with STH: STH/CH1MC3 (**A**), STH/CH2MC2 (**B**), and STH/CH2MC3 (**C**).

**Figure 5:**
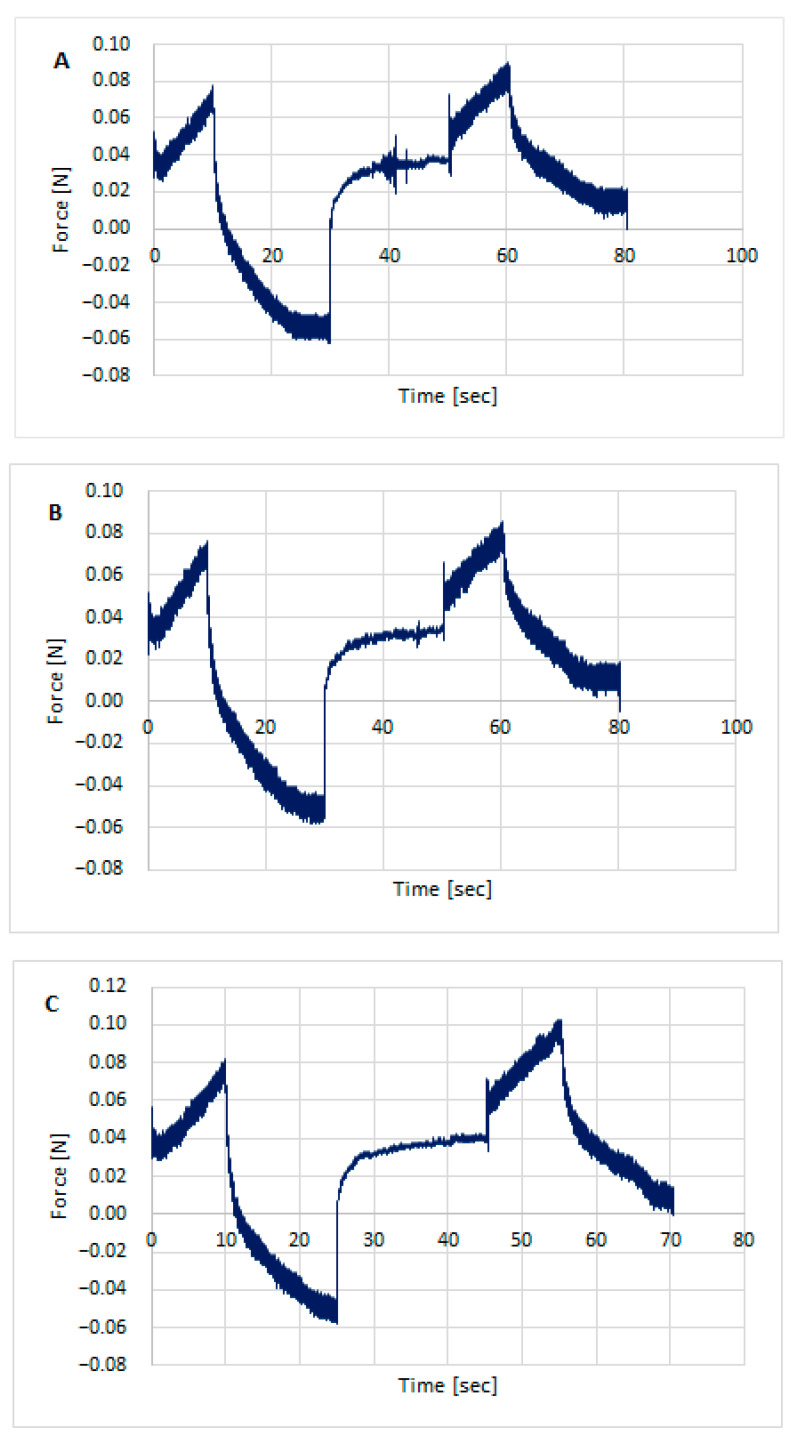
Texture profile analysis (TPA) of hydrogels with STH and PV: STH/CH1MC3 + PV 0.005 (**A**), STH/CH1MC3 + PV 0.0075 (**B**), and STH/CH1MC3 + PV 0.01 (**C**).

Texture testing was performed on the prepared hydrogel formulations using TPA Cycle and CRT. Figure 3, Figure 4 and Figure 5 show the distribution of forces acting on the samples during the double compression and tension test. The parameters determined using TPA and CRT—hardness, adhesive force, cohesion, adhesion, elasticity, and relaxation—are summarized in Table 6. The transdermal system must have favorable properties, such as good skin spreadability, optimal viscosity, ease of removal from the packaging, and good adhesion. Texture profile analysis is used to gather information about the semisolid dosage form, such as the mechanical properties of the gel, which include compressibility, hardness, and adhesion. A work-time plot is used to determine the spreadability of the formulation. The area under the curve is a measure of the force required to deform the sample a given distance. Adhesion is defined as the work required to overcome the attractive forces between the sample and probe surfaces [57]. Toughness is defined as the resistance to maximum compression during the initial compression cycle. Compressibility, i.e., the work required to deform the sample during the first compression of the probe, is calculated from the area under the force-time curve 1 (AUC1). Adhesiveness, the work required to overcome the attractive forces between the sample surface and the probe surface, is denoted as the negative force area for the first compression cycle and calculated from AUC2. Cohesion is defined as the ratio of the positive force area of the second compression cycle to the force area of the initial compression cycle, where the two compressions are separated by a defined recovery period. Elasticity is the ratio of the time required to reach maximum structural deformation during the second compression cycle to the time generated during the first compression cycle [58,59].

All formulations exhibited good textural properties, as indicated by the parameters presented in Table 6: hardness, adhesion strength, cohesion, adhesion, elasticity, and relaxation. The differences in these parameters result from the different amounts of polymers comprising the matrix for the active ingredient, somatotropin. The hardness of the formulations ranged from 0.051 ± 0.001 for STH/CH2MC2 to 0.082 ± 0.001 for STH/CH1MC3 and STH/CH1MC3 + PV 0.01. Increasing the amount of chitosan from 1% to 2% had virtually no effect on the hardness parameter, while increasing the methylcellulose content from 2% to 3% resulted in a significant increase in this parameter. The incorporation of STH into the CH2MC2 hydrogel base did not result in an increase in hardness, whereas a pronounced increase in this parameter was observed for formulations prepared using the CH1MC3 and CH2MC3 bases. This increase may also be due to increasing the methylcellulose concentration to 3%. The incorporation of PV to the STH formulations slightly reduced the hardness values of all CH1MC3-based STH formulations.

STH increased the cohesion of the hydrogel formulations, while PV, conversely, reduced the cohesion. The decrease in cohesion was greater with increasing PV concentration. STH exhibited no substantial impact on the adhesion of the hydrogel formulations. Conversely, PV demonstrated a marked enhancement in adhesion, ranging from 0.3 to 0.4, across all hydrogels, irrespective of the amount of PV incorporated. This increase in adhesion was accompanied by a modest reduction in elasticity. The higher adhesive strength and adhesion compared to the base gels indicate greater adhesion of the STH and PV gels. This can be attributed to the presence of protein components, which also increase viscosity. These properties allow for predicting better retention on the skin compared to the hydrogel formulation without STH and PV.

All prepared hydrogels exhibited similar degrees of relaxation, a measure of how quickly a system returns to equilibrium after a disturbance. The texture analyzer examines the material’s response to specific forces, such as compression, tension, or shear, for a specified time. After a certain deformation is reached, the force is held constant, and the device measures how the stress in the sample changes. Over time, the stress in the sample decreases, a phenomenon known as relaxation. This decrease in stress is measured and analyzed. The relaxation parameter allows us to assess how quickly a material loses its stress. Materials with strong viscoelastic behavior exhibit significant stress relaxation, while stiffer materials will exhibit less relaxation. All STH, STH, and PV formulations, as well as the base media, exhibited good relaxation, ranging from 74.2 ± 1.25% (STH/CH1MC3 + PV 0.0075) to 79.4 ± 1.27% (STH/CH2MC2).

The parameters of texture analysis can be associated with rheological properties and the extent of somatotropin release in a release study. An increase in the viscosity of the formulations was observed, along with their mechanical strength. The higher the viscosity of the hydrogels, the lower the drug retention through the cellulose membrane and the lower the bioavailability. All analyzed properties indicate that the prepared formulations can be applied to the skin, spread easily, and adhere to the skin surface, ensuring STH penetration over a sufficiently long period and slow release of the active ingredient.

### 3.4. Values of pH

Table 7 presents the pH values of the prepared formulations. Variations in pH values are visible, depending on the content of individual polymers. Increasing the CH concentration from 1% to 2% resulted in an increase in pH. Increasing the MC content from 2% to 3% did not significantly change the pH. STH reduced the pH to 5.06 for STH/CH1MC3 but only slightly increased the pH in the remaining formulations from 5.88 (CH2MC2) to 5.95 (STH/CH2MC2) and from 5.90 (CH2MC3) to 5.98 (STH/CH2MC3). A clear relationship was observed between PV concentrations and pH value—the higher the PV concentration, the lower the pH value. However, all tested formulations were acidic and ranged between 5.05 (STH/CH1MC3 + PV 0.01) and 5.98 (STH/CH2MC3), which is close to the natural skin pH (pH 4.5–6.5). It was observed that STH slightly increased the pH in hydrogels containing 2% chitosan but decreased it in hydrogels containing 1% chitosan. Methylcellulose did not cause such changes. Depending on the concentration, PV also slightly increased the pH of the STH-loaded hydrogel (concentrations 0.005% and 0.0075%), while at the highest concentration (0.01%) it had virtually no effect on the pH of the STH/CH1MC3 formulation. All formulations fell within the range of 4.5–6.5, making them safe for skin use without risk of irritation.

### 3.5. Sensory Evaluation of Prepared Hydrogels 

Based on the sensory testing (Table 8) of all prepared hydrogel preparations, it was found that they were characterized by a high degree of uniformity and very good adhesion to the skin surface. Additionally, they exhibited low stickiness and excellent application properties, enabling easy and even spreading.

## 4. Discussion

Hydrogels represent a unique class of semi-solid systems that exhibit solid-like behavior under static conditions. This physicochemical characteristic ensures excellent adhesion at the site of application and effectively prevents sedimentation. Under mechanical stress, however, hydrogels undergo a transition to a liquid-like state, enabling precise and uniform application to the skin surface. Local drug delivery via hydrogel-based systems offers several significant therapeutic advantages. Compared with conventional routes of administration, topical application avoids undesirable degradation in the gastrointestinal tract, loss of biological activity associated with the first-pass effect, and uncontrolled pharmacokinetic variability [61,62].

Methylcellulose (MC), when used as a carrier for protein-based substances, facilitates their penetration through the skin. A hydrogel matrix containing 6% MC released somatotropin (STH) within 4 h, achieving a cumulative release of 71.44% [22]. In contrast, a formulation containing 4% MC prolonged the STH release time to 7 h; however, the cumulative amount of STH released decreased to 46.68% [19].

MC exhibits several physicochemically advantageous properties, including temperature-induced gelation, surface activity at the air–water interface, and adsorption at the solid–water interface. These cellulose derivatives are water soluble and display amphiphilic behavior due to the methylation of the hydroxyl (–OH) groups of anhydro-D-glucose units. A degree of substitution exceeding 1.3, combined with a heterogeneous distribution of methyl groups, promotes the formation of hydrophobic domains driven by hydrophobic interactions. These domains play a critical role in thermogelation, a phenomenon that has been exploited in the design of transdermal drug delivery systems. Below a critical temperature threshold (29 ± 2 °C), the viscosity of aqueous MC solutions remains constant or decreases slightly with increasing temperature, reflecting the temperature sensitivity of MC [63].

The combination of polymers is a well-established approach for tailoring functional and application-related properties of hydrogel systems. In CH/MC-based formulations, MC primarily acts as a structural scaffold, while CH enhances cross-linking and adhesion, thereby improving gelation properties and reducing the gelation temperature of the system [64,65]. The resulting hydrogels form hydrophobic interactions and lower interfacial tension, which facilitates the retention of thermosensitive components within the polymer network [66,67].

Swelling behavior and structural integrity are critical parameters for hydrogel performance, as water absorption supports nutrient transport, cellular infiltration, and water retention [68,69]. A CH/MC hydrogel with a 2%/4% polymer ratio demonstrated particularly favorable physicochemical properties, including high viscosity and skin adhesion, leading to enhanced tissue retention and sustained insulin release (39.8% after 7 h), as well as effective penetration through the stratum corneum [54].

In the present study, artificial membranes were utilized in release experiments as a standard preformulation tool to assess the effect of hydrogel composition on drug release kinetics prior to skin permeation studies. Cellulose membranes, which are available in a range of pore sizes, ensure reproducible conditions and allow permeability to be matched to the molecular size of the compound. The absence of enzymes and lipids in these samples enables the evaluation of purely physical diffusion without protein degradation. Their structural homogeneity facilitates direct comparison between formulations, while the lack of significant interactions with somatotropin or polymers prevents interference with diffusion. While cellulose membranes do not replicate the epidermal barrier, they facilitate the estimation of the maximum release potential of the active substance. This capacity is especially advantageous in the early formulation stages.

Franz diffusion cells equipped with a 100 kDa dialysis membrane were used in the STH release study. This system is designed to evaluate the rate of drug release from hydrogels intended for topical application, under in vitro conditions. The method reflects the combined processes of STH release from the hydrogel and its diffusion through the dialysis membrane.

In this context, release refers to the liberation of the active substance from the hydrogel matrix and occurs independently of any biological barrier. It allows determination of the rate and extent of active substance release from the formulation. Such studies are typically performed using dialysis membranes in Franz diffusion cells, which do not constitute a biological barrier but serve only to separate the donor and receptor phases.

In contrast, permeation refers to the transport of the active substance across a biological barrier, such as the skin, and takes into account the properties of the barrier, including the stratum corneum. Permeation studies allow assessment of whether and to what extent the substance penetrates the skin. In the next stage of the study, STH permeation will be evaluated using porcine skin under ex vivo conditions.

The release study demonstrated that the highest cumulative amount of STH was released from the STH/CH2MC2 hydrogel (87.28 ± 4.27%), followed by STH/CH1MC3 (83.05 ± 8.39%), while the lowest release was observed for STH/CH2MC3 (71.26 ± 1.80%). The STH/CH2MC2 formulation exhibited the lowest viscosity, which likely facilitated a higher extent of STH release. In this formulation, complete STH release was achieved within the shortest time (t = 4 h), whereas STH release from the STH/CH1MC3 and STH/CH2MC3 hydrogels occurred within 5 h. This observation may indicate that a higher methylcellulose (MC) content contributes to prolongation of the release process.

The use of phosvitin (PV) as a carrier for STH markedly extended the release duration from 5 h to at least 10 h. At the highest PV concentration (0.01%), a delayed release profile was also observed. The cumulative amount of STH released was dependent on PV concentration, with higher PV levels resulting in lower STH release. The most pronounced effect was observed for PV at a concentration of 0.005%, yielding the highest cumulative STH release (91.84 ± 4.85%).

The release of STH from hydrogels intended for dermal administration represent a complex process. For STH-loaded hydrogels without PV, the release profiles were best described by a first-order kinetic model. The coefficients of determination (R^2^) for the STH/CH1MC3, STH/CH2MC2, and STH/CH2MC3 formulations were highest for this model, indicating that the release rate was proportional to the amount of STH remaining within the matrix and decreased over time. This behavior suggests a release mechanism governed by concentration-dependent kinetics, likely resulting from a combination of diffusion and a progressive reduction in the concentration gradient. Such first-order kinetics are characteristic of hydrogel systems with relatively simple diffusion profiles and without pronounced swelling or structural rearrangements.

In contrast, formulations containing PV were more accurately described by the Peppas–Sahlin and Higuchi models; however, higher R^2^ values were obtained for the Peppas–Sahlin model. This model provided an excellent fit to the experimental data and indicates the contribution of two concurrent mechanisms: Fickian diffusion and polymer relaxation or swelling. The incorporation of phosvitin appears to shift the release mechanism toward relaxation- or swelling-controlled transport rather than simple diffusion, thereby prolonging STH release. These findings are consistent with previously reported studies [70,71,72,73]. The Higuchi model, which assumes diffusion-controlled release without polymer degradation, further supports the role of the hydrogel matrix structure in governing STH transport [74,75].

Chitosan did not significantly affect the release kinetics of STH compared with hydrogels based solely on methylcellulose. In formulations containing 4% or 6% MC, STH release followed first-order kinetics. In contrast, the incorporation of carrier proteins such as phosvitin or albumin has been shown to markedly modify STH release behavior. As demonstrated in earlier studies, the incorporation of PV into a 4% MC hydrogel has been shown to extend the duration of STH permeation through porcine skin from four hours to a minimum of seven hours. The formulation containing 0.001% PV has been observed to yield the most effective sustained release, a phenomenon that can be most accurately described by the Korsmeyer–Peppas model [46]. The study of the STH release from a 6% MC-based hydrogel demonstrated first-order release kinetics, both in vitro using artificial cellulose membranes and under simulated in vivo conditions with porcine skin. STH availability exceeded 80%, while albumin prolonged STH release to at least 24 h and increased availability up to 93%, depending on its concentration [22].

The rheological characterization of the developed hydrogels confirmed their favorable application properties, ensuring sufficient retention on the skin and supporting prolonged drug release. The ideal transdermal hydrogel should demonstrate viscosities ranging from 1 to 100 Pa·s at low shear rates (0.1–1 s^−1^), thereby ensuring sufficient residence time. Furthermore, pseudoplastic behavior is advantageous, as it ensures high viscosity at rest—enhancing formulation stability—and reduced viscosity under shear, facilitating easy application and uniform spreading [61,76,77].

Higher chitosan concentrations resulted in a reduced amount of STH released during the release studies. A greater amount of STH was released from the STH/CH1MC3 formulation compared with STH/CH2MC3. However, this trend was not observed for the STH/CH2MC2 formulation, which exhibited the highest STH availability. In this case, the increased availability may be attributed to the lower MC content in the hydrogel matrix, suggesting that MC exerts a stronger influence on the release profile than chitosan. This observation further confirms the role of rheological properties in modulating STH release. The higher viscosity, hardness, and cohesion of the STH/CH1MC3 and STH/CH2MC3 hydrogels relative to STH/CH2MC2 likely account for their lower release rates, while simultaneously contributing to greater rheological stability.

In the CH1MC3, CH2MC2, and CH2MC3 formulations, increasing the chitosan (CH) content from 1% to 2% resulted in a slight decrease in viscosity, whereas increasing the methylcellulose (MC) content from 2% to 3% led to a moderate increase in viscosity. These observations indicate that MC contributes more strongly to viscosity enhancement than CH. The incorporation of STH into the base hydrogels increased viscosity. In contrast, the introduction of phosvitin (PV) reduced the viscosity of STH-containing formulations, which may improve their spreadability on the skin. This viscosity reduction is likely due to altered intermolecular interactions. Although protein solutions often show increased viscosity from electrostatic effects, certain additives can decrease viscosity with increasing concentration, probably through hydrophobic interactions and protein conformational changes that modify rheological behavior [78].

All CH/MC-based hydrogels exhibited pseudoplastic, shear-thinning behavior and thixotropy, reflecting gradual disruption and recovery of the internal network under shear. Higher thixotropy indices indicated greater rheological stability, supporting the suitability of these formulations as carriers for dermal drug delivery [79].

The flow behavior index values were correlated with the degree of pseudoplasticity, with lower values indicating more pronounced shear-thinning behavior. The tested hydrogels exhibited relatively low flow indices (0.105–0.448), which is indicative of a well-ordered internal structure. Higher flow index values correspond to less structurally ordered systems. In general, the flow behavior index decreases from values close to unity, characteristic of Newtonian fluids, to values between 0 and 1, typical of non-Newtonian systems. This transition can be explained by increased polymer chain mobility and a reduced lifetime of polymer chain aggregates under shear stress [55].

Amaral Sobral et al. demonstrated that chitosan solutions at various concentrations and temperatures exhibit non-Newtonian (pseudoplastic) behavior, with viscosity decreasing as shear rate increases due to gradual microstructure disruption and dominant biopolymer–biopolymer interactions [80]. Conversely, dilute chitosan solutions (≤0.5%) exhibited near-Newtonian behavior, suggesting predominant biopolymer–water interactions [81]. Abu-Jdayil et al. reported analogous observations, finding non-Newtonian flow for chitosan concentrations between 0.1% and 3%, with the exception of ≤0.5% concentrations, where reduced chain entanglement led to Newtonian behavior [82,83].

In CSDA–MC (decanoic acid–modified chitosan) hydrogels prepared at various polymer ratios, viscosity decreased under shear and recovered upon shear removal, reflecting reversible disruption and reformation of hydrogen bonds between polymers and water. This shear-dependent structural recovery has been demonstrated to support good spreadability, ease of application, and adhesion to the application site [84].

Texture analysis of the prepared hydrogel formulations included the determination of parameters such as hardness, adhesive strength, cohesion, adhesion, elasticity, and relaxation. The maximum force recorded during the test was adopted as a measure of hardness, with higher values indicating harder samples [85]. Hardness reflects the mechanical resistance of the formulations and, in the investigated systems, ranged from 0.051 ± 0.001 N to 0.084 ± 0.002 N. Adhesive force and tack values (approximately 0.4 ± 0.07 mJ for STH formulations containing PV) ensure adequate adherence of the formulation to the application site and an appropriate residence time on the skin, which is essential for achieving the desired therapeutic effect.

Texture analysis was utilized in conjunction with rheological evaluation, thereby facilitating a comprehensive assessment of the functional properties of the developed hydrogel matrices. This approach also served to support further optimization of the formulation. The area under the positive portion of the force–time curve obtained during texture profile analysis (TPA) reflects sample consistency, with higher values indicating thicker formulations. Cohesion, defined as the ability of the gel to regain its structural integrity after deformation, is a critical factor in determining its performance at the application site. Higher cohesion has been shown to enhance the effectiveness of the gel in these settings. The maximum negative force is frequently utilized as an indicator of cohesiveness, with more negative values corresponding to stronger structural integrity.

Consistent with previous reports, increased cohesion has been observed in mucoadhesive gels containing liposome-encapsulated drugs [86]. All investigated formulations, including STH- and STH/PV-containing systems as well as base vehicles, exhibited satisfactory stress relaxation (74.2–79.4%), indicating adequate stability during storage and application and supporting the preservation of STH bioactivity.

Rheological, viscoelastic, and bioadhesive properties are increasingly important for topical and transdermal formulations, which should exhibit pseudoplastic behavior to form a coherent film on the skin and ensure prolonged residence time at the application site [79,87]. Increased viscosity (e.g., higher MC content) favors prolonged peptide release, whereas reduced viscosity (e.g., after PV incorporation) improves spreadability but may decrease matrix resistance. Therefore, an appropriate balance between viscosity and structural integrity is essential for modulating STH release kinetics.

The physicochemical properties of the developed hydrogels were suitably optimized to achieve the intended release profile. The prolonged release obtained with PV confirms its potential as a transport protein in preformulation studies. The integrated analysis of rheological, textural, and release characteristics presented herein provides a basis for the rational design of polysaccharide-based hydrogels with tailored drug release profiles [88].

The assay of the active pharmaceutical ingredient (API) is a critical quality attribute of pharmaceutical formulations. The acceptable specification limits are 90–110% of the declared content in the United States [50] and 95–105% in Europe [89]. In the present study, these requirements were met both immediately after hydrogel preparation and after 4 weeks of storage.

The skin’s tolerance to topical formulations is closely related to the pH level of the formulations. In order to ensure optimal skin compatibility and to minimize the potential for irritation, it is essential that formulations maintain a pH level within the physiological range of the skin. The prepared hydrogels exhibited slightly acidic pH values (5.05–5.98), consistent with normal skin pH (4.5–6.5), indicating good dermatological tolerability [90,91,92].

The incorporation of STH and PV resulted in only minor pH changes, which remained within the acceptable range. An increase in PV concentration resulted in a gradual decrease in pH, while an increase in chitosan content from 1% to 2% led to a slight increase in pH, likely due to reduced protonation of chitosan as the pH approached its pKa (~6.5). Hydrogels with higher pH values exhibited lower viscosity, consistent with reduced electrostatic repulsion between polymer chains. Conversely, lower pH promoted increased protonation, chain extension, and higher viscosity [93,94,95].

This phenomenon has been documented in the extant literature. Cid et al. [96] observed a slight increase in pH with increasing chitosan concentration (1–3%). It has been demonstrated in other studies that the pH of the chitosan solution is contingent upon concentration and formulation conditions, manifesting as either an increase or a decrease in pH [80]. Chitosan–methylcellulose hydrogels have been reported to display a broad pH range (4.0–7.0) at 37 °C, allowing the selection of formulations with tailored physicochemical properties for dermatological applications [97].

Sensory evaluation showed that all hydrogels exhibited uniform dispersion, no phase separation, no air bubbles, and exhibited adequate adhesion with minimal viscosity and easy spreadability. These properties are crucial for ensuring formulation stability, long-term persistence on the skin, therapeutic efficacy, and user comfort during topical application.

## 5. Conclusions

The release profile of somatotropin (STH) was influenced more strongly by the methylcellulose content than by the chitosan content, with the rheological properties of the hydrogel matrix playing a crucial role in modulating the release kinetics. Among the investigated formulations, STH/CH2MC2 exhibited the highest STH availability, whereas STH/CH2MC3 showed the lowest. All hydrogel systems demonstrated non-Newtonian, pseudoplastic, and thixotropic behavior, confirming their adequate rheological stability and favorable application properties.

The incorporation of proteinaceous components, namely somatotropin (STH) and phosvitin (PV), enhanced the adhesion and mechanical resistance of the hydrogels. Phosvitin functioned as an effective carrier, significantly prolonging STH release from the hydrogel matrix for up to 10 h. The formulation containing 0.005% PV achieved the highest STH release, while a higher PV concentration (0.01%) resulted in a more pronounced delay and extension of the release process.

Protein-loaded hydrogels intended for topical application offer several advantages, including controlled and prolonged drug release, reduced dosing frequency, and avoidance of degradation associated with oral administration. Therefore, the developed hydrogel systems represent promising candidates for the design of sustained-release formulations capable of maintaining relatively stable therapeutic drug levels.

Further research is warranted to confirm these findings. Future studies should include in vitro permeation experiments using porcine skin, investigations in appropriate animal models (e.g., rats or rabbits), and ultimately clinical studies in humans. Previous work by the authors involving simulated in vivo permeation models has indicated that porcine skin exhibits higher permeability than that predicted by preliminary studies using artificial cellulose membranes. Consequently, in vivo investigations represent a critical next step in the preformulation development of STH-containing hydrogel systems.

## Figures and Tables

**Table 1:** Composition of the prepared hydrogel formulations.

Ingredient	CH [g]	0.1 M Acetic Acid [g]	MC [g]	Glycerol [g]	Water [g]	STH [g]	PV [g]
CH1MC3	1.0	49.0	3.0	2.0	45.0	-	-
CH2MC2	2.0	48.0	2.0	2.0	46.0	-	-
CH2MC3	2.0	48.0	3.0	2.0	45.0	-	-
STH/CH1MC3	1.0	49.0	3.0	2.0	44.9	0.1	-
STH/CH2MC2	2.0	48.0	2.0	2.0	45.9	0.1	-
STH/CH2MC3	2.0	48.0	3.0	2.0	44.9	0.1	-
STH/CH1MC3 + PV 0.005	1.0	49.0	3.0	2.0	44.895	0.1	0.005
STH/CH1MC3 + PV 0.0075	1.0	49.0	3.0	2.0	44.8925	0.1	0.0075
STH/CH1MC3 + PV 0.01	1.0	49.0	3.0	2.0	44.89	0.1	0.01

**Table 2:** Area under the curve (AUC), release rate, and determination coefficients R^2^ for the STH preparations.

Preparation		STH/CH1MC3	STH/CH2MC2	STH/CH2MC3
AUC _[0-n]_		196.28 ± 16.25 ^2^	243.23 ± 11.55 ^1^	172.59 ± 4.44 ^1^
Release rate [mg/cm^2^ h^−1^]		0.087 ± 0.010 ^2^	0.103 ± 0.004 ^1^	0.075 ± 0.004
Release rate constant		0.374 ± 0.012	0.412 ± 0.036	0.249 ± 0.012 ^1^
R^2^	Higuchi	0.9341	0.9711	0.9388
	Kormeyer-Peppas	0.9320	0.9678	0.9254
	First-order	0.9613	0.9876	0.9601
	Peppas-Sahlin	0.9080	0.9613	0.9170
Preparation		**STH/CH1MC3** **+ PV 0.005**	**STH/CH1MC3** **+ PV 0.0075**	**STH/CH2MC3** **+ PV 0.01**
AUC _[0-n]_		552.28 ± 43.22 ^1^	491.76 ± 5.09 ^1^	471.62 ± 49.73 ^1,2^
Release rate [mg/cm^2^ h^−1^]		0.047 ± 0.001 ^1^	0.043 ± 0.002 ^1,2^	0.042 ± 0.004 ^1^
R^2^	Higuchi	0.9952	0.9953	0.9974
	Kormeyer-Peppas	0.9585	0.9923	0.9256
	First-order	0.9830	0.9876	0.9840
	Peppas-Sahlin	0.9960	0.9967	0.9980

^1^—statistically significant difference with respect to the STH/CH1MC3 preparation. ^2^—statistically significant difference with respect to the STH/CH1MC3 + PV 0.005 preparation

**Table 4:** Viscosity of the prepared hydrogels determined at 32 °C; mean and SD from 6 replicates.

Preparation	Viscosity η (mPa·s)		
	30 s^−1^	60 s^−1^	100 s^−1^
CH1MC3	13,674.6 ± 117.1	8828.8 ± 120.5	6520.0 ± 96.2
CH2MC2	12,080.8 ± 120.3 ^1^	7945.9 ± 102.1 ^1^	6001.1 ± 78.3 ^1^
CH2MC3	12,378.3 ± 116.3 ^1^	8234.1 ± 97.6 ^1^	6256.1 ± 59.5 ^1^
STH/CH1MC3	19,097.1 ± 321.6 ^1^	12,023.5 ±147.2 ^1^	8790.9 ± 69.5 ^1^
STH/CH2MC2	14,440.5 ± 119.1 ^2^	9052.4 ± 89.5 ^2^	6905.2 ± 74.1 ^2^
STH/CH2MC3	18,173.8 ± 105.9	10,762.2 ± 101.2 ^2^	7611.6 ± 93.2 ^2^
STH/CH1MC3 + PV 0.005	16,663.3 ± 316.8 ^2^	10,258.8 ± 187.3 ^2^	7924.2 ± 152.9 ^2^
STH/CH1MC3 + PV 0.0075	15,134.7 ± 284.8 ^2^	9725.9 ± 129.9 ^2^	7145.9 ± 136.6 ^2^
STH/CH1MC3 + PV 0.01	17,388.3 ± 350.2 ^2^	11,048.8 ± 219.8 ^2^	8056.2 ± 176.1 ^2^

^1^—statistically significant difference with respect to the CH1MC3 base. ^2^—statistically significant difference with respect to the STH/CH1MC3 preparation.

**Table 5:** The results were obtained from mathematical modeling of the rheograms. Parameters calculated by fitting equations Ostwald-de Waele, Bingham, Herschel–Bulkley, and Casson.

Hydrogel	Ostwald			Bingham		Herschel–Bulkley				Casson	
	*n*	K	R^2^	τ_0_	R^2^	τ_0_	*n*	K	R^2^	τ_0_	R^2^
CH1MC3	0.225	358.8	0.989	662.0	0.911	0.05	0.244	305.5	0.993	438.0	0.950
CH2MC2	0.141	651.8	0.961	986.0	0.828	0.02	0.134	667.4	0.978	841.0	0.913
CH2MC3	0.175	632.4	0.943	1081	0.902	0.02	0.175	632.4	0.976	867.0	0.836
STH/CH1MC3	0.328	161.8	0.993	397.4	0.918	0.05	0.328	161.8	0.993	253.1	0.965
STH/CH2MC2	0.105	889.7	0.981	1204	0.831	0.05	0.105	889.7	0.981	1066	0.922
STH/CH2MC3	0.448	81.9	0.984	293.9	0.923	0.05	0.448	81.9	0.984	151.0	0.957
STH/CH1MC3 + P 0.005	0.287	216.2	0.987	492.3	0.895	0.05	0.287	216.2	0.987	336.6	0.950
STH/CH1MC3 + P 0.0075	0.354	149.6	0.988	404.3	0.931	0.05	0.354	149.6	0.988	248.1	0.966
STH/CH1MC3 + P 0.01	0.290	235.9	0.987	541.0	0.886	0.05	0.290	235.9	0.987	366.5	0.951

Symbols: τ_0_, yield stress [Pa]; K, consistency index [Pa·s^n^]; *n*, flow behavior index; R^2^, determination coefficient.

**Table 6:** Texture parameters of the tested formulations: mean and standard deviation (n = 6).

Preparation	Hardness [N]	Adhesion Force [N]	Cohesiveness	Adhesiveness [mJ]	Elasticity	Relaxation [%]
CH1MC3	0.057 ± 0.0	−0.044 ± 0.001	2.057 ± 0.004	0.3 ± 0.0	0.715 ± 0.001	75.86 ± 0.84
CH2MC2	0.059 ± 0.003	−0.037 ± 0.001 ^1^	1.781 ± 0.047 ^1^	0.3 ± 0.05	0.750 ± 0.011 ^1^	72.70 ± 1.39 ^1^
CH2MC3	0.081 ± 0.001 ^1^	−0.057 ± 0.001 ^1^	1.983 ± 0.033 ^1^	0.4 ± 0.05	0.697 ± 0.002 ^1^	75.25 ± 0.64 ^1^
STH/CH1MC3	0.082 ± 0.001 ^1^	−0.058 ± 0.0 ^2^	2.777 ± 0.057 ^1^	0.3 ± 0.0	0.640 ± 0.012 ^1^	75.77 ± 1.65
STH/CH2MC2	0.051 ± 0.001 ^2^	−0.032 ± 0.001 ^2^	3.009 ± 0.062 ^2^	0.2 ± 0.0 ^2^	0.530 ± 0.003 ^2^	79.4 ± 1.273 ^2^
STH/CH2MC3	0.078 ± 0.002 ^2^	−0.058 ± 0.001 ^2^	2.083 ± 0.058 ^2^	0.4 ± 0.0 ^2^	0.692 ± 0.002 ^2^	75.38 ± 1.56 ^2^
STH/CH1MC3 + PV 0.005	0.078 ± 0.002 ^2^	−0.062 ± 0.004 ^2^	2.469 ± 0.030 ^2^	0.4 ± 0.07 ^2^	0.52 ± 0.001 ^2^	77.4 ± 0.95
STH/CH1MC3 + PV 0.0075	0.076 ± 0.002 ^2^	−0.058 ± 0.010 ^2^	2.278 ± 0.040 ^2,3^	0.4 ± 0.07 ^2^	0.52 ± 0.0 ^2^	74.2 ± 1.25 ^2,3^
STH/CH1MC3 + PV 0.01	0.082 ± 0.001 ^2,3^	−0.058 ± 0.0 ^2^	2.133 ± 0.040 ^2^	0.4 ± 0.07 ^2^	0.69 ± 0.040 ^3^	74.60 ± 0.75 ^3^

^1^—statistically significant difference with respect to the CH1MC3-based hydrogel. ^2^—statistically significant difference with respect to the STH/CH1MC3 preparation. ^3^—statistically significant difference with respect to the STH/CH1MC3 + P0.005 preparation.

**Table 7:** pH values of prepared hydrogels (mean with standard deviation; n = 6).

Hydrogel Preparation Code	Average pH Value and Standard Deviation
CH1MC3	5.39 ± 0.010
CH2MC2	5.88 ± 0.014 ^1^
CH2MC3	5.90 ± 0.017 ^1^
STH/CH1MC3	5.06 ± 0.001 ^1^
STH/CH2MC2	5.95 ± 0.016 ^2^
STH/CH2MC3	5.98 ± 0.008 ^3^
STH/CH1MC3 + PV 0.005	5.26 ± 0.006 ^4^
STH/CH1MC3 + PV 0.0075	5.15 ± 0.012 ^4^
STH/CH1MC3 + PV 0.01	5.05 ± 0.001 ^4^

^1^—statistically significant difference compared to the CH1MC3 vehicle. ^2^—statistically significant difference compared to the CH2MC2 vehicle. ^3^—statistically significant difference compared to the CH2MC3 vehicle. ^4^—statistically significant difference compared to the STH/CH1MC3 hydrogel.

**Table 8:** Parameters determined in the sensory evaluation of the prepared hydrogel preparations [60].

Parameter	CH1 MC3	CH2 MC2	CH2 MC3	STH/ CH1MC3	STH/ CH2MC2	STH/ CH2MC3	STH/ CH1MC3 + PV 0.005	STH/ CH1MC3 + PV 0.0075	STH/ CH1MC3 + PV 0.01
Uniformity	5	5	5	5	5	5	5	5	5
Consistency	3	2	3	3	2	3	2	3	3
Cushioning effect	3	2	2	3	2	2	2	2	2
Adhesion	5	4	5	4	4	4	4	4	4
Spreadability	5	5	5	5	5	5	5	5	5
Stickiness	2	2	2	2	2	2	2	2	2
Greasiness and oiliness	1	1	1	1	1	1	1	1	1

Uniformity 1—visible stratification, lumps, and bubbles, 5 no lumps or air bubbles. Consistency 1—no resistance when dipping, 5—resistance when dipping and lifting the finger is similar. Cushion effect 1—no cushion effect, 5—significant cushion effect. Adhesion 1—very low adhesion, 5—very high, distinct, stringy cone. Spreadability 1—difficult to spread, 5—very easy to spread. Stickiness 1—slight stickiness, 5—significant stickiness. Greasiness and oiliness—1 imperceptible greasiness, 5—significant greasiness.

**Table 3:** Similarity and Difference Coefficients F2 and F1.

Comparable Preparations	F1	F1	F2	F2
STH/CH1MC3 vs. STH/CH1MC3 + PV 0.005	128.21	dissimilar	188.63	dissimilar
STH/CH1MC3 vs. STH/CH1MC3 + PV 0.0075	133.50	dissimilar	5.7	dissimilar
STH/CH1MC3 vs. STH/CH1MC3 + PV 0.01	138.72	dissimilar	3.03	dissimilar
STH/CH1MC3 + PV 0.005 vs. STH/CH1MC3 + PV 0.0075	13.7	similar	42.83	dissimilar
STH/CH1MC3 + PV 0.005 vs. STH/CH1MC3 + PV 0.01	15.68	dissimilar	33.09	dissimilar
STH/CH1MC3 + PV 0.0075 vs. STH/CH1MC3 + PV 0.01	6.67	similar	54.97	similar
STH/(CH1MC3) vs. STH/(CH2MC2)	36.96	dissimilar	179.87	dissimilar
STH/(CH1MC3) vs. STH/(CH2MC3)	12.45	similar	169.41	dissimilar
STH/(CH2MC2) vs. STH/(CH2MC3)	44.43	dissimilar	189.26	dissimilar

## Data Availability

The original contributions presented in this study are included in the article/supplementary material. The data presented in this study are available upon request. The author (corresponding author) can provide them.

## Supplementary Materials

The following supporting information can be downloaded at: https://www.mdpi.com/article/10.3390/polym18010086/s1, Table S1: Viscosity of the prepared hydrogels determined at 25 °C, mean and SD from 6 replicates; Table S2: Viscosity of the prepared hydrogels determined at 32 °C after 4 weeks storage, mean and SD from 6 replicates; Table S3: Texture parameters of the tested formulations after 4 weeks storage at 4 °C; mean and standard deviation (n = 6); Table S4: pH values of prepared hydrogels after 4 weeks (mean with standard deviation; n = 3).
