# Supplementary material for: Evaluation of Somatropin Release from Chitosan and Methylcellulose Hydrogels: Influence of Hydrogel Composition and Phosvitin on the Release Profile
Source: Polymers (Basel). 2025 Dec 28;18(1):86. doi: 10.3390/polym18010086 (PMC12787833; doi:10.3390/polym18010086)
Supplement: Supplementary file 1 [file polymers-18-00086-s001.zip › polymers-4028646-supplementary.pdf]

Table S1. Viscosity of the prepared hydrogels determined at 25 °C, mean and SD from 6 replicates.

Preparation	Viscosity η (mPa·s)		
	**30 s⁻¹**	**60 s⁻¹**	**100 s⁻¹**
CH1MC3	14905.3 ± 284.6	9358.5 ± 167.5	6846.7 ± 178.6
CH2MC2	13047.3 ± 129.3 [1]	8462.4 ± 146.5 [1]	6301.2 ± 169.7 [1]
CH2MC3	13616.1 ± 216.8 [1]	8892.8 ± 150.2 [1]	6631.5 ± 153.5 [1]
STH/CH1MC3	21006.8 ± 147.5 [1]	12865.1 ± 179.7 [1]	9054.6 ± 156.9 [1]
STH/CH2MC2	15884.6 ± 108.3 [2]	9686.1 ± 106.2 [2]	7250.5 ± 179.4 [2]
STH/CH2MC3	19991.2 ± 116.9	11730.8 ± 123.3 [2]	7839.9 ± 98.7 [2]
STH/CH1MC3+PV 0.005	17996.3 ± 136.5 [2]	10976.9 ± 113.6 [2]	8161.9 ± 101.5 [2]
STH/CH1MC3+PV 0.0075	16345.5 ± 207.5 [2]	10406.7 ± 184.7 [2]	7360.3 ± 168.9 [2]
STH/CH1MC3+PV 0.01	19127.1 ± 196.2 [2]	12043.2 ± 104.6 [2]	8297.9 ± 173.9 [2]

[1] - statistically significant difference with respect to the CH1MC3 base.

[2] - statistically significant difference with respect to the STH/CH1MC3 preparation.

Table S2. Viscosity of the prepared hydrogels determined at 32 °C after 4 weeks storage, mean and SD from 6 replicates.

Preparation	Viscosity η (mPa·s)		
	**30 s⁻¹**	**60 s⁻¹**	**100 s⁻¹**
CH1MC3	13576.5 ± 149.9	8943.1 ± 105.5	6606.3 ± 87.3
CH2MC2	12217.9 ± 117.1 [1]	8035.1 ± 114.1 [1]	6059.6 ± 76.4 [1]
CH2MC3	12491.6 ± 105.9 [1]	8376.7 ± 81.2 [1]	6305.7 ± 99.8 [1]
STH/CH1MC3	19262.2 ± 114.3 [1]	12150.8 ± 97.1 [1]	938.6 ± 87.9 [1]
STH/CH2MC2	14570.9 ± 147.4 [2]	8963.1 ± 67.9 [2]	6954.1 ± 83.6 [2]
STH/CH2MC3	17911.8 ± 120.5	10960.4 ± 72.9 [2]	7684.8 ± 97.5 [2]
STH/CH1MC3+PV 0.005	16517.7 ± 144.7 [2]	10346.1 ± 88.5 [2]	7999.6 ± 68.8 [2]
STH/CH1MC3+PV 0.0075	15385.3 ± 113.4 [2]	9835.4 ± 77.9 [2]	7039.4 ± 79.6 [2]
STH/CH1MC3+PV 0.01	17629.5 ± 188.1 [2]	11168.3 ± 91.4 [2]	7976.8 ± 85.1 [2]

[1] - statistically significant difference with respect to the CH1MC3 base

[2] - statistically significant difference with respect to the STH/CH1MC3 preparation

Table S3. Texture parameters of the tested formulations after 4 weeks storage at 4 °C; mean and standard deviation (n=6).

Preparation	Hardness [N]	Adhesion force [N]	Cohesiveness	Adhesiveness [mJ]	Elasticity	Relaxation [%]
CH1MC3	0.057 ± 0.01	-0.045 ± 0.003	2.107 ± 0.014	0.3 ± 0.03	0.703 ± 0.001	75.78 ± 1.84
CH2MC2	0.059 ± 0.017	-0.036 ± 0.012 [1]	1.821 ± 0.027 [1]	0.3 ± 0.03	0.759 ± 0.013 [1]	72.14 ± 1.12 [1]
CH2MC3	0.082 ± 0.012 [1]	-0.057 ± 0.004 [1]	1.993 ± 0.033 [1]	0.4 ± 0.05	0.678 ± 0.003 [1]	75.87 ± 0.46 [1]
STH/CH1MC3	0.082 ± 0.01 [1]	-0.058 ± 0.007 [2]	2.542 ± 0.048 [1]	0.3 ± 0.02	0.652 ± 0.012 [1]	75.64 ± 1.16
STH/CH2MC2	0.050 ± 0.005 [2]	-0.032 ± 0.010 [2]	2.954 ± 0.049 [2]	0.2 ± 0.0 [2]	0.527 ± 0.011 [2]	79.45 ± 1.24 [2]
STH/CH2MC3	0.079 ± 0.012 [2]	-0.058 ± 0.001 [2]	2.183 ± 0.037 [2]	0.4 ± 0.0 [2]	0.684 ± 0.002 [2]	75.58 ± 1.36 [2]
STH/CH1MC3+ PV 0.005	0.078 ± 0.008 [2]	-0.062 ± 0.004 [2]	2.545 ± 0.038 [2]	0.4 ± 0.05 [2]	0.538 ± 0.001 [2]	77.39 ± 1.15
STH/CH1MC3+ PV 0.0075	0.077 ± 0.013 [2]	-0.057 ± 0.010 [2]	2.249 ± 0.026 [2,3]	0.4 ± 0.08 [2]	0.545 ± 0.01 [2]	74.31 ± 1.10 [2,3]
STH/CH1MC3+ PV 0.01	0.083 ± 0.007 [2,3]	-0.056 ± 0.007 [2]	2.147 ± 0.049 [2]	0.4 ± 0.08 [2]	0.675 ± 0.014 [3]	74.74 ± 0.98 [3]

[1] - statistically significant difference with respect to the CH1MC3-based hydrogel

[2] - statistically significant difference with respect to the STH/CH1MC3 preparation

[3] - statistically significant difference with respect to the STH/CH1MC3+P0.005 preparation

Table S4. pH values of prepared hydrogels after 4 weeks (mean with standard deviation; n=3).

Hydrogel Preparation Code	Average pH value and standard deviation
CH1MC3	5.41 ± 0.015
CH2MC2	5.9 ± 0.004 [1]
CH2MC3	5.88 ± 0.008 [1]
STH/CH1MC3	5.07 ± 0.01 [1]
STH/CH2MC2	5.98 ± 0.002 [2]
STH/CH2MC3	5.98 ± 0.009 [3]
STH/CH1MC3+PV 0.005	5.27 ± 0.006 [4]
STH/CH1MC3+PV 0.0075	5.16 ± 0.003 [4]
STH/CH1MC3+PV 0.01	5.07 ± 0.011 [NS]

[1] - statistically significant difference compared to the CH1MC3 vehicle

[2] - statistically significant difference compared to the CH2MC2 vehicle

[3] - statistically significant difference compared to the CH2MC3 vehicle

[4] - statistically significant difference compared to the STH/CH1MC3 hydrogel
